# Example food: What are its sensory properties and why is that important?

**DOI:** 10.1038/s41538-018-0019-3

**Published:** 2018-06-19

**Authors:** Herbert Stone

**Affiliations:** Sensory Consulting Services, Menlo Park, CA USA

**Keywords:** Human behaviour, Technology

Today’s marketplace for food and beverages is diverse and competitive in ways not imagined a few decades ago. It is driven, in part, by consumers and consumer blogs asking questions about the safety of ingredients and processed foods, in general. Some of these questions are about GMOs and/or the need for gluten free, organic, and natural foods. It also has led to the development of products by non-traditional food companies that offer alternatives vs. foods currently available. Regardless of the product, source of raw materials, and potential benefits offered, success is determined ultimately by the consumer. History tells us that a unique ingredient or a food formulated that meets some or all of the aforementioned characteristics does not guarantee market success. Although there are numerous steps between development and consumption by the consumer where a product can fail, its sensory properties are critical in establishing its potential and continued success.

Where product success is achieved, changes happen reflecting the dynamics of the marketplace and include such issues as the shift to larger scale production, availability and cost of raw materials, competition, and so forth. Any change results in a different product and management needs to know whether the change is important, i.e., is it recognized by the consumer and does it change purchase intent? What product characteristics or combination of characteristics have changed? What can technology do to compensate if this change has negative effects? Answers to these kinds of questions are best obtained through use of sensory evaluation, a science that has long been used for the evaluation of products but not as well-known as other consumer testing resources, e.g., market research.

## What is sensory evaluation?

Sensory evaluation is a science that measures, analyzes, and interprets the reactions of people to products as perceived by the senses. It is a means of determining whether product differences are perceived, the basis for the differences, and whether one product is liked more than another. The value of the science lies in its use of limited numbers of consumers to reach decisions that can be extrapolated to larger populations with confidence. This means that the subjects are representative of the consumer population for whom the product is intended and have the necessary sensory skills. In practical terms, it enables one to evaluate products in a relatively short time and at low cost. In the discussion that follows, key sensory resources are described; however, a detailed discussion about the science can be found in the reference list at the end of this article.

## Resources

There are four types of resources that serve as the basis for the science. These are subjects, methods, test plans and analyses, and facilities and support services. Each is summarized below.

### Subjects

Sensory evaluation relies on consumers to provide the data on which decisions are based. Although there are consumers everywhere to provide responses, there are considerable differences in any population. They look different and not surprisingly, their sensory skills are different, by as much as 100% or more in sensitivity to differences among products. Depending on the type of information needed, analytical or affective, different skill levels or qualifications are required.

For analytical methods (discrimination and descriptive), subjects need to be above average users of a product/product category and demonstrate ability to discriminate differences at better than chance among those products.

Empirical evidence from more than 50 years of testing around the world has shown that about 30% of any population cannot discriminate differences at better than chance. This is independent of age, frequency of product use, gender, and other typical criteria. It means that those who do not meet this requirement should not participate as sensitivity is decreased and variability is greater. Otherwise, there is a high risk of concluding there is no difference when, in fact, there is (β risk). For more on risk, see Cohen^1^.

Qualifying subjects for analytical tests is a straightforward process taking about 4–5 h in a series of three or four 90 min test sessions. Consumers have to learn how to use their senses, how to take a test, and familiarize themselves with the products. Based on numerous experiments, it was concluded that having a consumer participate in about 30–40 difference test trials was necessary before determining an individual is qualified. It is surprising how often this process is ignored and recommendations based on test results are not supported in the marketplace. See Stone et al.^2^^,^^3^ for a more extensive discussion on this topic.

For affective tests, i.e., liking or preference, the key qualification is product and brand use. Above average product use is necessary, as those consumers are generally found to be more sensitive than the infrequent user.

### Methods

There are many test methods available for use; all have specific applications and can be categorized as analytical or affective.

**Fig. 1:**
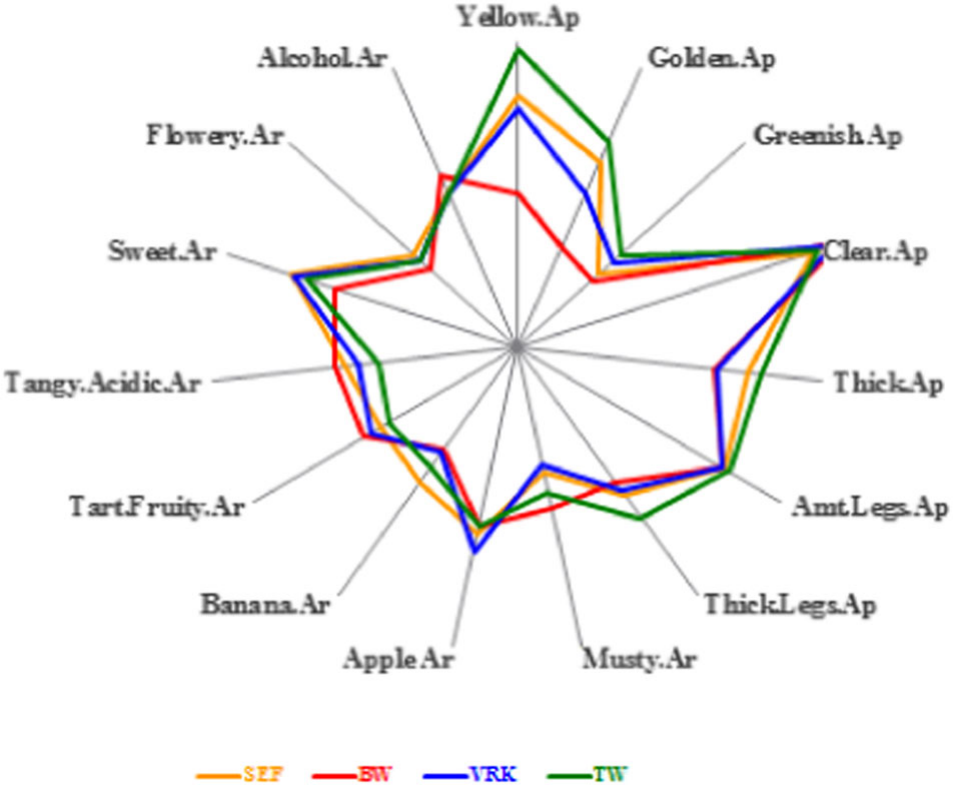
A sensory map of aroma and appearance attributes obtained from a descriptive analysis of an competitive chardonnay wines. The intensity values are measured from the center to that point where a line crosses and are the computed means obtained from a panel of 12 subjects and 4 replicates (*N* = 48)

Analytical methods include discrimination/difference and descriptive analysis. Results from a difference test tell us whether product differences are perceived. Results from a descriptive test identify the specific kinds of differences, e.g., a fruit aroma for a series of samples and their intensities. An example of results from the latter is shown in Fig. 1, a sensory map showing results from a test of competitive wines.

Analytical tests rely on limited numbers of qualified subjects but all require replication. This is no different than a chemical analysis of a product which is done more than once to have confidence in the reported result. For discrimination, between 20 and 30 subjects are recommended. With replication this yields sufficient judgments to have confidence in the conclusions. More discussion about this recommendation, see Stone^2^.

For descriptive analysis, the optimal number of subjects is 12 and not < 10. Empirically, it has been observed that using more than 12 does not result in more information, only greater statistical significances. Using fewer than 10 makes it more difficult to obtain expected significances. With a 6-product test, 30–40 attributes (typical for foods and beverages), and 4 replicates for each product, there are sufficient data to enable a thorough analysis of subject skill and specific product differences.

Affective methods include hedonic and preference. Results from a hedonic test tell us the degree of liking for series of products. It is used primarily for determining which product is best liked from an array of options. Preference methods determine which product is preferred. About 75–100 subjects are recommended for an affective sensory test.

No sensory method is more sensitive than another. The choice of method is based on the test objective and the nature and number of products to be tested. If products have a lingering taste, then methods requiring multiple sampling within a test session would not be appropriate because of sensory fatigue.

A few comments are warranted here about the use of the internet as a means for obtaining information from consumers. Various methods have been described in the literature and have attracted considerable attention. One can collect large amounts of information in a relatively short time period; a process no different than surveys using landlines to recruit for a test or measure attitudes about social or political issues. One of the advantages today is the ability to provide pictures and descriptions of products which add focus to the consumer’s responses. In early stage development of ideas, this approach has value to brand managers and technical staff; however, without knowledge of a person’s sensory skills, it does not, as yet, appear to be a substitute for more traditional sensory procedures.

### Test plans and analyses

The importance of using appropriate design and analysis cannot be underestimated. Plans must reflect behavioral and physiological elements in the design which means that balanced designs are preferred and designs that rely on randomized serving orders should be avoided. Otherwise, risk is high that one sample will be served in one position several times, whereas another product is never served in that position. First sample effects cannot be eliminated so one must be sure that each product appears equally often in each position. Although this is relatively easy to achieve in a test of four or fewer products, it is more difficult in a test of ten products. A solution is to balance the design across all subjects. With replication, the serving order challenges are minimized but not eliminated. A variety of test designs have been published while others are connected with software packages; however, all need to be reviewed before use.

In situations where products are scored, a common feature of descriptive analysis and liking, the most useful analysis is the analysis of variance. As with any statistical procedure, it is important that the data collection process fit the model on which the test plan is based. Much has been written about statistical methods and the interested reader is directed to Stone^2^.

### Facilities and support services

Traditionally, sensory tests were fielded in a purpose built facility with a controlled environment and partitions to minimize visual contact between subjects and servers. Responses were captured on paper ballots and then transcribed for analysis. Today’s facility has touch screens and the ability to provide real time analyses regardless of where the data were obtained. In addition this has enabled the actual data collection to no longer be restricted to a sensory facility. Use of a tablet and the web has made it possible to test products anywhere, based on the purpose for the information. This means that managers can obtain information and transmit instructions to others regardless of their location.

## Application, an example

As previously noted, a sensory map is very useful in identifying the effects of formulation changes on sensory attributes from a developer’s perspective. Maps also have value for marketing and brand managers when results are connected with preferences and other consumer measures.

**Fig. 2:**
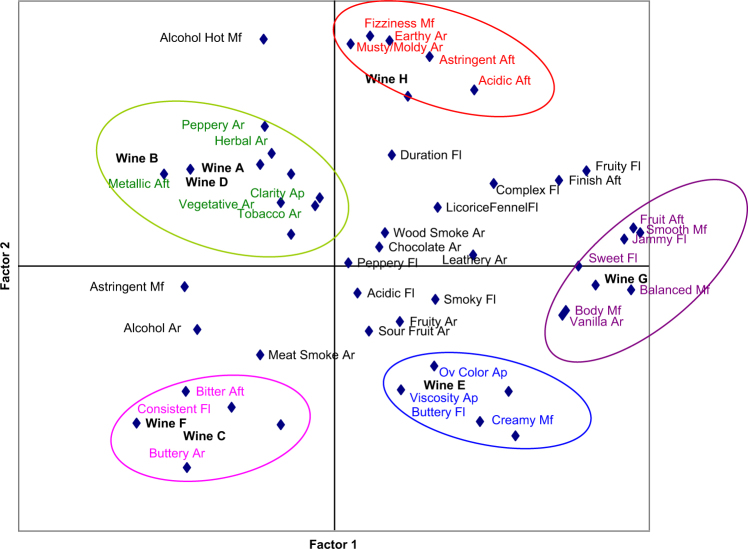
A bi-plot of the sensory attributes and preferences obtained from wine consumers. The attributes that are most associated with specific wines are circled

Figure 2 shows results from a plot of sensory attributes and consumer preferences. The circled groupings show the connection between specific sensory attributes and preferences enabling marketing to identify which word groupings are best used in communicating to which consumers. Additional discussion about this and other applications are described in the aforementioned references.

## Conclusion

Sensory evaluation is all about people using their senses vs. simply eating a meal without appreciating the experience. Learning to use one’s senses has many rewards especially as it relates to a greater appreciation for the food and beverages we consume. Companies invest substantial sums of money in developing and marketing products without fully appreciating the consumer’s ability to identify what makes one product a success and another just getting by. Sensory information should be an integral part of any product effort, whether at the formulation stage or at the marketing stage if one expects to be more successful in satisfying consumer expectations.
